# Development and Initial Validation of a Brief, Online Version of the Center for Epidemiological Studies Depression Scale (CES-D): Psychometric Study

**DOI:** 10.2196/81595

**Published:** 2026-01-08

**Authors:** Moshe Shmueli, Ariel Pollock Star, Nofar Tsur, Norm O'Rourke

**Affiliations:** 1 Goldman Medical School Faculty of Health Sciences Ben-Gurion University of the Negev Be'er Sheva Israel; 2 Department of Epidemiology, Biostatistics, and Community Health Sciences Ben-Gurion University of the Negev Be'er Sheva Israel; 3 Department of Science, Technology and Society Bar-Ilan University Ramat Gan Israel; 4 Department of Psychology Faculty of Humanities and Social Sciences Ben-Gurion University of the Negev Be'er Sheva Israel; 5 Center for Multidisciplinary Research in Aging Faculty of Health Sciences Ben-Gurion University of the Negev Be'er Sheva Israel

**Keywords:** Center for Epidemiological Studies Depression Scale, CES-D, depression, social media use, social support, structural equation modeling

## Abstract

**Background:**

A growing volume of mental health research is conducted with participants recruited and responding online. However, to date, few psychometric scales have been specifically validated for online research.

**Objective:**

We aimed to devise a brief, 12-item version of the Center for Epidemiological Studies Depression Scale (CES-D) in which first order factors are sufficiently measured.

**Methods:**

We recruited 218 adults with depression and 226 comparison participants with no mental health history. Both groups completed the original 20-item CES-D and measures of social support, psychological distress, and sociodemographic information (eg, age, gender, and household income). Measurement of social support included online support, and psychological distress included symptoms of social media use disorder along with loneliness and life dissatisfaction.

**Results:**

This brief, 12-item version of the CES-D was devised with persons with depression and replicated with comparison participants. For both, core sadness, somatic symptoms, interpersonal detachment, and absence of well-being each significantly contributed to measurement of a higher-order depression latent construct (*P*<.01). Structural equation modeling was performed to establish the construct validity of this 4-factor model in which depression is predicted by socioeconomic factors and depression predicts lower social support as well as greater psychological distress.

**Conclusions:**

Responses to this 12-item, online version of the CES-D demonstrate factorial and construct validity. Clinical research is required in future to ascertain whether scores greater than 11 (of 36) are suggestive of elevated depressive symptomology.

## Introduction

### Background

As many facets of modern life are moving online, so too is mental health research [1]; this includes both participant recruitment and data collection [2]. According to King et al [3], this evolution of research methodology has had a democratizing effect, broadening recruitment beyond psychiatric hospitals and outpatient clinics and enabling contact with subpopulations who eschew biomedical interventions [4]. However, comparatively few psychometric scales have been validated specifically for online research. This paper describes the development of a brief online version of the Center for Epidemiological Studies Depression Scale (CES-D). The factor and construct validity of this online CES-D was tested relative to social support (including online social support) and psychosocial distress (including symptoms of social media use [SMU] disorder).

Our intent was to devise a brief version of the CES-D based on the original version that measures of each of the 4 factors identified across populations and over time [5]. This includes the interpersonal factor, which is currently measured using just 2 items; however, latent constructs should be measured by 3 or more items [6]. Due to the primary role of social relations and support in depression [7], this factor requires adequate measurement.

### Clinical Depression

According to the *Diagnostic and Statistical Manual of Mental Disorders, Fifth Edition, Text Revision* [8], depression is characterized by persistent low mood; loss of interest or pleasure in activities (ie, anhedonia); and various physical, cognitive, and behavioral symptoms. In addition, it has become increasingly clear in recent years that interpersonal factors are central to the experience of clinical depression [9]. A growing body of research underscores the importance of social networks, loneliness, and social support in relation to depression across populations [9-11].

Developed nearly 50 years ago [12], the CES-D remains a widely used screening tool and measure of depressive symptomology [13,14]. The original CES-D consists of 20 items [15] to which respondents indicate how each applies to how they have felt over the previous week. Different cutoff values have been suggested [5], yet few clinical or sociodemographic differences appear to affect the reliability of CES-D responses [16]. Over time, various brief versions of the CES-D have been proposed [17].

Factor analyses of CES-D responses have consistently identified 4 first-order factors mapping onto a higher-order latent construct [18]. That is, depression is not a singular phenomenon but a multidimensional construct composed of both physical and cognitive symptoms (cf insomnia vs sadness). The 4 first-order factors measured by the CES-D are core sadness (eg, “I had crying spells”), absence of well-being (eg, “I felt hopeful about the future”; reversed), somatic symptoms (eg, “My sleep was restless”), and interpersonal rejection (eg, “I felt that people dislike me”).

Emerging research supports the existence of SMU disorder as a bona fide psychiatric condition associated with clinical depression [19], though findings are inconsistent [20]. As a means for connection, online platforms such as Facebook and Instagram can shape perceptions and either foster or negate social connectedness. In its original form, the CES-D may not fully or efficiently measure the phenomenology of depression. Through the creation of a terse version of the scale but with interpersonal detachment more fully measured, the field will have an abridged CES-D specifically devised for online research.

For this study, we hypothesized that responses from persons with and without depression would be reliable (eg, internal consistency) and normally distributed. For both, we hypothesized that each CES-D factor would significantly contribute to measurement of a higher-order depression latent construct. Structural equation modeling (SEM) was used to demonstrate the construct validity of the responses to this brief CES-D relative to psychological distress, sociodemographic status, and traditional and online social support.

## Methods

### Recruitment

Adults with and without a diagnosis of clinical depression were recruited as part of a larger study on SMU and well-being. Participants had to be aged ≥18 years and able to read English. Both groups were recruited using the Positly platform. Positly has previously been used to recruit participants for both psychometric [21,22] and mental health research [3,21,22]. For this study, only those who had previously reported a depression diagnosis were asked to complete the depression questionnaire; similarly, participants in the comparison sample had to have reported no psychiatric history. We also excluded comparison participants who provided CES-D responses above the clinical threshold (ie, CES-D score >15) as possibly having depression but currently undiagnosed.

Most Positly participants are Amazon Mechanical Turk contractors who have established themselves as reliable over many piecework “gigs” in the crowdsourcing marketplace [23]. Over time and multiple projects, participants provide more descriptive information (ie, psychiatric diagnoses) to take part in better-paying gigs. Positly excludes participant responses (ie, not paid) if descriptive information contradicts that previously provided or if questionnaire responses are mostly missing or determined to be unreliable (ie, rapid responding without reading the questions). Positly is designed to work with the Qualtrics questionnaire platform (Qualtrics International Inc). Anonymized sociodemographic information is provided by Positly (eg, age and household income) along with questionnaire responses.

### Instruments

The original CES-D [12] is composed of 20 items with responses reported on a Likert scale ranging from *rarely or none* (0) to *most or all the time* (3). The CES-D has been translated into more than a dozen languages [24].

As first noted more than 35 years ago [18], CES-D responses reflect a complex structure with correlated factors, each measuring a higher-order depression latent construct [16]. This has been demonstrated in cross-national research with representative samples [25].

Although other solutions have been proposed [26], the 4-factor structure first proposed by Hertzog et al [18] has been widely replicated across populations [25,27,28]. These first-order factors have been labeled as depressive affect (eg, “I thought my life had been a failure”), absence of well-being (eg, “I felt hopeful about the future”), somatic symptoms (eg, “I did not feel like eating; my appetite was poor”), and interpersonal rejection (eg, “I felt that people dislike me”). Scale scores greater than 15 out of 60 on the CES-D are suggestive of clinically significant depressive symptomatology [29].

The Brief University of California, Los Angeles (UCLA-8) Loneliness Scale [30] is an abbreviated measure developed to identify associations between loneliness and health-related behaviors [31]. Responses are reported on a Likert scale ranging from *I never feel this way* (0) to *I often feel this way* (3). Internal consistency of UCLA-8 responses is high (Cronbach α=0.84) [32].

The Satisfaction With Life Scale (SLS) [33] measures perceived quality of life based on person-specific criteria [34]. The SLS is composed of 5 questions (eg, “The conditions of my life are excellent”) with response alternatives ranging from *strongly disagree* (1) to *strongly agree* (7). Test-retest reliability over 1 month has been reported as *r*=0.84 [34]. The concurrent validity of SLS responses has been demonstrated relative to the Fordyce global scale (*r*=0.82) [34].

The Brief Social Support Scale [35] measures perceived support, divided between emotional-informational and tangible support. Responses to each of the 6 items are reported on a Likert scale ranging from *very dissatisfied* (1) to *very satisfied* (6). The reliability of the responses to both subscales is high (Cronbach α=0.87 and 0.86, respectively) [35].

The Online Social Support Scale [36] is a 40-item measure of subjective online social support that divides support into 4 domains: instrumental, informational, esteem or emotional, and social companionship. Responses are reported on a Likert scale from *never* (0) to *a lot* (4). Responses have shown strong internal consistency (Cronbach α=0.97) [37], yet this high Cronbach α value may suggest item redundancy [6].

The Social Media Disorder Scale (SMD) [38] operationalizes excessive or pathological SMU as disordered or addicted behavior based on the proposed diagnostic criteria for internet gaming disorder (*Diagnostic and Statistical Manual of Mental Disorders, Fifth Edition, Text Revision*) [8]. Responses to each of the 9 items are dichotomously reported (ie, “yes” or “no”). SMD total scores greater than 5 are suggestive of clinically problematic SMU [38]. SMD responses have good internal consistency (Cronbach α=0.81) [38].

A sociodemographic questionnaire was constructed for this study to obtain descriptive, psychosocial, and clinical information [39,40]. Participants reported date of birth, gender, country of residence, relationship status, level of education, occupation, and ethnicity. Both those with and without depression were also asked to list all diagnosed mental health conditions, approximate dates of diagnosis, comorbid psychiatric conditions, prescribed psychotropic medications, and alternate therapies. To verify the correct categorization of both persons with depression and comparison participants, we corroborated diagnostic information.

### Statistical Analyses

We first performed multiple imputation to address missing scale responses using the *mice* package in R (R Foundation for Statistical Computing) [41]. Missing or skipped responses were less than 1% of scale responses and missing at random (ie, not specific to particular items). In part, this low percentage was due to the prompting function in Qualtrics requiring respondents to confirm that they intended to skip responses before proceeding to the next question. Descriptive statistics were first computed for both continuous dependent and independent variables to confirm that their distributions were relatively normal and had good internal consistency.

Confirmatory factor analysis (CFA) was conducted to examine responses to the original 4-factor CES-D. A revised 12-item model was proposed to enhance measurement of the interpersonal rejection latent factor. This model was independently replicated with participants with depression and those with no mental health history.

SEM was performed to confirm the construct validity of the responses to this revised CES-D. We hypothesized that each first-order CES-D factor would map onto a higher-order depression latent construct; in turn, depressive symptomatology was assumed to be predicted by social support and sociodemographic variables, and depressive symptomatology and social support were assumed to predict psychological well-being.

In accord with convention, we report 3 goodness-of-fit indexes to assess overall model fit for both CFA and SEM models: an incremental (comparative fit index; CFI), an absolute (standardized root mean square residual; SRMR), and a parsimonious (root mean square error of approximation; RMSEA) fit index. Ideal SRMR and RMSEA values are less than 0.055, and ideal CFI values are greater than 0.95 [6].

We also report the expected cross-validation index (ECVI) to compare distinct models. Unlike other goodness-of-fit statistics, there is no defined cutoff value for the ECVI; instead, lower values suggest greater likelihood of replication in the population than larger values [42]. Like with the RMSEA, 90% CIs are estimated for the ECVI.

Bootstrapping was also performed as a computationally intensive simulation approach to obtain parameter estimates in the population with this model. Bootstrapping treats the original sample as a pseudopopulation or stand-in for population data [43]. Multiple random samples are then drawn from the original sample along with replacement values; each is used to obtain a new set of parameter estimates. The distribution of these parameter estimates is used to compute bias-corrected CIs for path coefficients. CIs that do not cross 0 are statistically significant and likely to exist in the population [43].

Descriptive and comparative analyses were performed using SPSS (version 29; IBM Corp); CFA, SEM, and invariance analyses were performed using SPSS AMOS (version 29; IBM Corp). SAS (SAS Institute) was used to estimate statistical power for CFA and SEM models.

### Ethical Considerations

Ethics approval was obtained from the institutional review board at Ben-Gurion University of the Negev, Be’er Sheva, Israel (499-7; August 2023). Respondents provided informed consent to participate by clicking to proceed as stated on the study splash page. They were not required to provide identifying information aside from an email address if they wished to receive a summary of the findings or take part in the participant lottery (US $500). All aspects of this study were performed in accordance with the Declaration of Helsinki.

## Results

### Descriptive Statistics

From March 2024 to October 2024, we recruited 444 adults in the United States via the Positly platform; 218 (49.1%) were recruited from a pool of persons who previously indicated that they had been diagnosed with depression, and we also recruited a comparison sample of 226 (50.9%) adults with no mental health history. Participants in both groups confirmed this categorization at recruitment.

On average, participants took 15.47 (SD 10.09) minutes to complete the online questionnaires; this did not significantly differ between groups (*t*_442_=0.26; *P*=.80). On average, participants were aged 45.88 (SD 13.13; range 22-79) years, yet the depression sample (mean age 44.56, SD 12.5 years) was somewhat younger than the comparison sample (mean age 47.25, SD 13.6 years; *t*_444_=2.22; *P*=.03). Consistent with depression prevalence, there were significantly more women in the depression sample (147/218, 67.4% women vs 69/218, 31.7% men) than in the comparison sample (98/226, 43.4% women vs 110/226, 48.7% men; *χ*^2^_3_=50.0; *P*<.01). Among participants with depression, time since diagnosis varied widely, with a mean of 14.98 (SD 11.03) years, ranging from 2 months to 66.34 years (average age 14.98, SD 11.03 years).

Only 50% (109/218) of the participants with depression were currently prescribed psychotropic medication; almost one-quarter (52/210, 23.85%) reported various alternate therapies (eg, meditation or marijuana). Of those taking prescribed medication, many were taking multiple medications, including antidepressants, mood stabilizers, anxiolytics, and atypical antipsychotics (mean number of prescribed psychotropic medications 0.66, SD 0.78; range 0-4).

Scores on the 20-item CES-D by persons with depression were high (ie, mean 25.51, SD 13.1), with 77.52% (169/218) obtaining scores suggestive of clinically elevated symptoms (ie, CES-D score >15). In contrast, the scores of comparison participants were considerably lower (mean 5.56, SD 4.34; *t*_444_=21.40; *P*<.01). Internal consistency was within ideal parameters for CES-D responses (Cronbach α=0.93 for both the depression and comparison groups) and all factors except interpersonal rejection (0.45<Cronbach α<0.71; Table 1).

**Table 1:** Descriptive statistics for study variables of participants with and without depression.

	Participants with depression (n=218)			Participants without depression (n=226)				*t* test (*df*)
	Mean (SD)	Cronbach α	Mean (SD)		Cronbach α			
Age (years)	44.56 (12.40)	—^a^	47.25 (15.35)			—	2.17 (444)^b^	
Education (years)	15.34 (7.87)	—	15.40 (2.96)			—	0.75 (434)	
Annual household income (US $)	71,985 (55,492)	—	77,724 (55,397)			—	1.09 (416)	
Original 20-item CES-D^c^	25.51 (13.10)	0.97	5.56 (4.34)			0.93	21.40 (444)^c^	
UCLA^d^ Loneliness Scale	20.44 (5.49)	0.93	13.26 (3.78)			0.77	16.03 (444)^c^	
Satisfaction With Life Scale	16.56 (7.89)	0.92	24.95 (7.16)			0.90	11.75 (444)^c^	
*S*ocial Media Disorder Scale	1.81 (1.97)	0.79	0.61 (1.17)			0.69	7.78 (444)^c^	
Tangible social support	8.35 (2.44)	0.90	9.88 (2.37)			0.92	6.73 (444)^c^	
Emotional social support	8.33 (2.54)	0.92	10.03 (2.14)			0.89	7.58 (444)^c^	
Online social support	30.39 (9.29)	0.92	32.60 (9.30)			0.94	2.50 (444)^c^	

^a^Not applicable.

^b^*P*<.05.

^c^CES-D: Center for Epidemiological Studies Depression Scale.

^c^*P*<.01.

^d^UCLA: University of California, Los Angeles.

### Original 20-Item CES-D

We first computed a CFA model examining responses by persons in the depression sample to the original 20-item version of the CES-D [12]. This CFA model was computed to generate comparative data for subsequent analyses. After correcting for correlated error between 9 of 210 possible item pairs, goodness of fit was within the ideal parameters for each statistic** **(*χ*^2^_158_=251.7; *P*<.01). More precisely, the CFI (0.96), SRMR (0.052), and RMSEA (0.052) were all within ideal parameters. The full 90% CI for the RMSEA was within adequate parameters (0.040<RMSEA CI 90% <0.064). The ECVI for this 20-item CES-D model was 1.64 (1.46<ECVI CI 90% <1.86).

Each CES-D item significantly loaded onto its respective first-order factor (ie, critical ratio values>|1.96|), though 3 of 20 items cross-loaded across 2 or more factors (ie, complex items). In addition, all first-order factors significantly contributed to measurement of a higher-order depression construct, including interpersonal rejection** **(β=0.73; *P*<.01). With 218 participants and 158 df, statistical power for this CFA model was sufficient to identify small effect sizes (ie, *d*>0.99) [6].

### 12-Item Online Version of the CES-D

**Figure 1:**
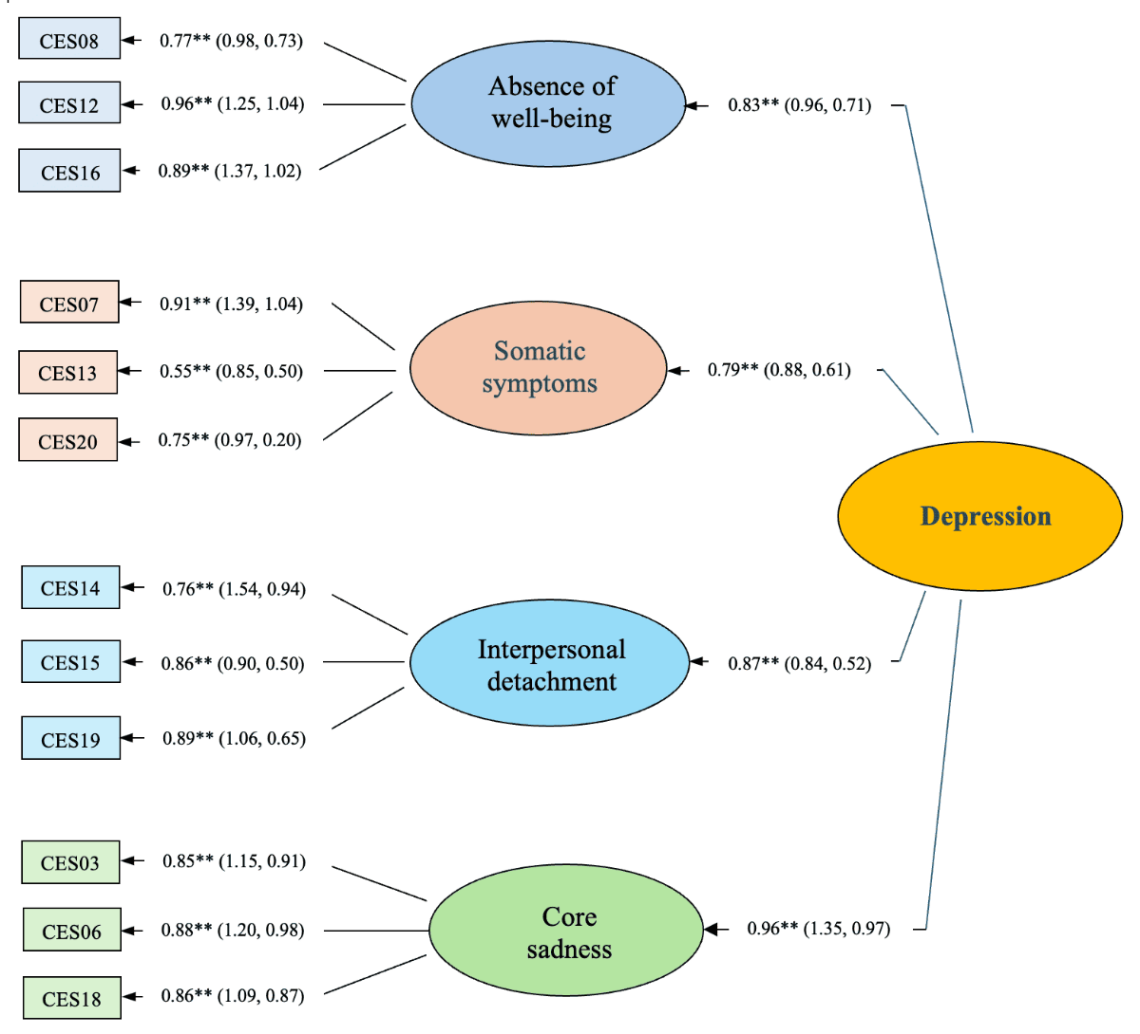
Brief, online version of the Center for Epidemiological Studies Depression Scale (CES-D). The image shows standardized β values (maximum likelihood estimates), with the 95% CIs in parentheses. The * indicates *P*<.05 and the ** indicates *P*<.01.

We next set out to identify a brief version of the CES-D with all factors measured using a minimum of 3 items. We first excluded all complex items, then identified those that loaded most significantly upon their respective factors (ie, lowest error estimates). This allowed us to determine whether we could expand measurement of the interpersonal factor by removing the loneliness item (16) from the sadness factor. On this basis, we provisionally relabeled this factor as *interpersonal detachment* rather than *interpersonal rejection* (Figure 1).

Again, all 12 items loaded significantly onto their respective first-order factors (critical ratio>|1.96|), and all factors were found to significantly measure a higher-order depression latent construct. This includes the expanded interpersonal detachment factor with a standardized β value of 0.87 (*P*<.01) compared to 0.73 (*P*<.01) for the 20-item CES-D. However, the increase was not statistically greater than the baseline model (Δ*χ*^2^=0.1; Δdf=1; *P*>.05), nor did measurement significantly differ for the other factors relative to the original 20-item model.

With the 12-item scale, correction for correlated error was required between items 15 and 19 (both on the interpersonal factor) to achieve ideal fit for all indexes (*χ*^2^_49_=72.0; *P*=.02). Thereafter, the CFI (0.99), SRMR (0.043), and RMSEA (0.046; 0.020<RMSEA 90% confidence level<0.068) were each within ideal parameters. With 218 participants and 49 df, this model had sufficient statistical power to detect small effect sizes (ie, *d*>0.99) [6].

As the 12-item model was nested in the 20-item CFA, we were able to compare relative fit (Δ*χ*^2^ = 251.7 – 72.0 = 179.7). This difference significantly exceeded the threshold value of 134.37 (ie, Δdf = 158 − 49 = 109; *P*<.05), suggesting a better fit for the revised 12-item CES-D vis-à-vis the original. The ECVI was also lower for the 12-item model than for the original (0.60 vs 1.64). Moreover, the 90% CIs did not overlap (0.51-0.72 vs 1.46-1.86), suggesting that a superior fit for the 12-item CES-D is likely to be found more than 90% of the time if replicated with samples of equal size.

### Replication of Findings for Persons Without Depression

We set out to replicate this 12-item, higher-order model with a comparison sample as the CES-D is administered as a screening measure to both persons with and without depression. Goodness of fit was again within the ideal parameters after correcting for correlated error between 2 item pairs (*χ*^2^_49_=69.6; *P*=.03; CFI=0.96; SRMR=0.052; RMSEA=0.043; 0.015<RMSEA 90% confidence level<0.065). With 226 participants and 49 df, this model also had sufficient statistical power to detect small effect sizes (ie, *d*>0.99 [6]; Table 2).

**Table 2:** Descriptive statistics for the revised 12-item Center for Epidemiological Studies Depression Scale (CES-D) for participants with and without depression.

		Participants with depression (n=218)		Participants without depression (n=226)		*t* test (*df*)
		Mean (SD)	Cronbach α	Mean (SD)	Cronbach α	
12-item online CES-D		16.60 (8.62)	0.92	3.53 (3.05)	0.69	21.14^a^
	Range of scores	0-36	—^b^	0-12	—	—
	1. Core sadness	4.27 (2.84)	—	0.44 (0.78)	—	19.19^a^
	2. Absence of well-being	5.18 (2.65)	—	1.72 (1.81)	—	18.69^a^
	3. Somatic symptoms	4.33 (2.43)	—	0.88 (1.24)	—	18.69^a^
	4. Interpersonal detachment	2.82 (2.29)	—	0.48 (0.83)	—	14.17^a^

^a^*P*<.01.

^b^Not applicable.

As expected, responses to this 12-item CES-D were strongly correlated with loneliness and inversely associated with life satisfaction. Moreover, brief CES-D responses were moderately and inversely associated with all forms or social support, including online social support. These results emerged for both participants with and without depression (Tables 3 and 4).

**Table 3:** Correlation coefficients between the 12-item Center for Epidemiological Studies Depression Scale (CES-D) and study variables for participants with depression.

		Tangible support	Emotional support	Online social support	Life satisfaction	Loneliness	Social media use disorder
**12-item CES-D**							
	*r*	−0.36	−0.49	−0.21	−0.64	0.71	0.19
	*P* value	.01	.01	.01	.01	.01	.01
**Tangible support**							
	*r*	1	0.57	0.31	0.36	−0.47	−0.04
	*P* value	—^a^	.01	.01	.01	.01	.52
**Emotional support**							
	*r*	0.57	1	0.37	0.49	−0.59	0.04
	*P* value	.01	—	.01	.01	.01	.52
**Online social support**							
	*r*	0.31	0.37	1	0.19	−0.19	0.25
	*P* value	.01	.01	—	.01	.01	.01
**Life satisfaction**							
	*r*	0.36	0.49	0.19	1	−0.61	−0.04
	*P* value	.01	.01	.01	—	.01	.60
**Loneliness**							
	*r*	−0.47	−0.59	−0.19	−0.61	1	0.13
	*P* value	.01	.01	.01	.01	—	.05

^a^Not applicable.

**Table 4:** Correlation coefficients between the 12-item Center for Epidemiological Studies Depression Scale (CES-D) and study variables for participants without depression.

			Tangible support		Emotional support		Online social support		Life satisfaction		Loneliness		Social media use disorder
**12-item CES-D**													
	*r*	−0.37		−0.38		−0.25		−0.39		0.53		0.20	
	*P* value	.01		.01		.01		.01		.01		.01	
**Tangible support**													
	*r*	1		0.72		0.23		0.34		−0.50		−0.06	
	*P* value	—^a^		.01		.01		.01		.01		.36	
**Emotional support**													
	*r*	0.72		1		0.32		0.33		−0.53		−0.09	
	*P* value	.01		—		.01		.01		.01		.52	
**Online social support**													
	*r*	0.23		0.32		1		0.14		−0.29		0.25	
	*P* value	.01		.01		—		.04		.01		.01	
**Life satisfaction**													
	*r*	0.34		0.33		0.14		1		−0.41		0.06	
	*P* value	.01		.01		.04		—		.01		.34	
**Loneliness**													
	*R*	−0.50		−0.53		−0.29		−0.41		1		0.13	
	*P* value	.01		.01		.01		.01		—		.05	

^a^Not applicable.

### Construct Validity of Responses to the 12-Item CES-D

**Figure 2:**
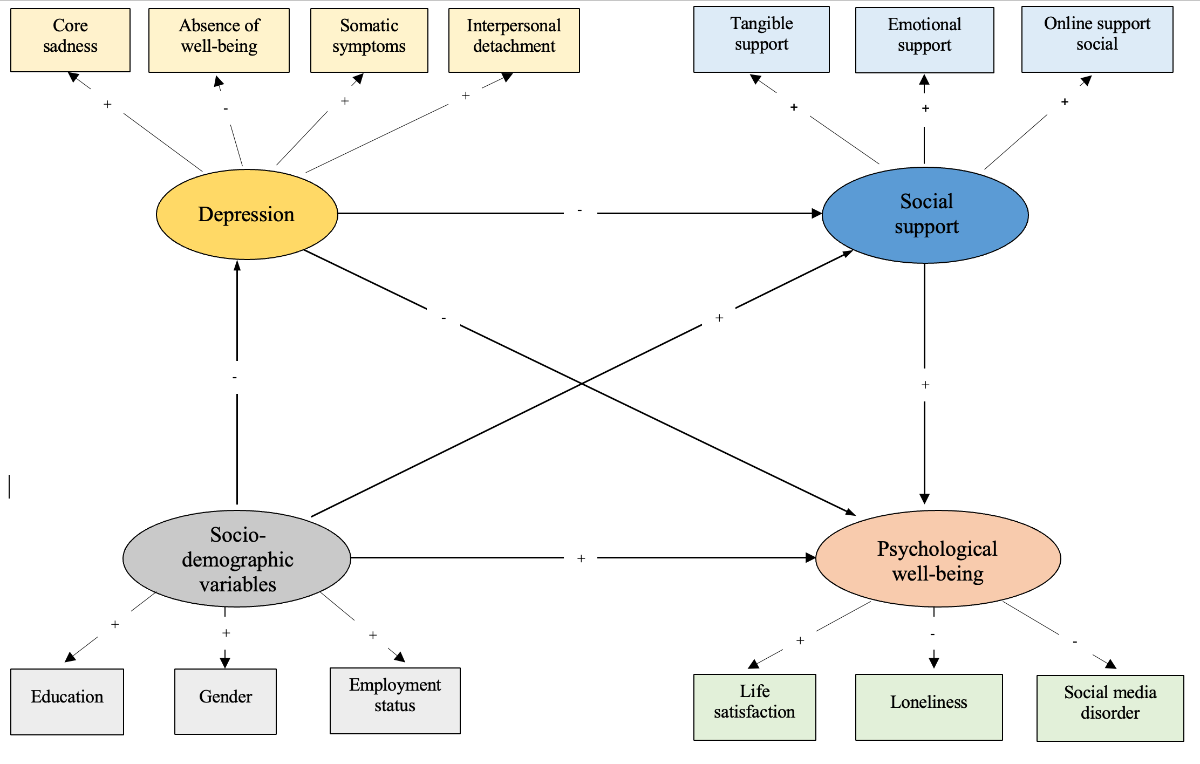
Hypothesized model; construct validity of 12-Item online Center for Epidemiological Studies Depression Scale (CES-D).

Having devised a brief version of the CES-D with all factors sufficiently measured, we next set out to confirm its construct validity relative to sociodemographic factors, social support, and psychological well-being. Using SEM, we hypothesized that depression as measured using these 4 factors would significantly predict lower social support and psychological well-being (Figure 2).

The resulting model emerged largely as hypothesized, yet neither gender nor employment status emerged as significant. Instead, we included household income and socioeconomic status based on work performed (now or before retirement). We relabeled this latent factor as socioeconomic status vs sociodemographic status. This suggests that depression is fostered by economic factors more than demographic factors (cf household income and gender).

**Figure 3:**
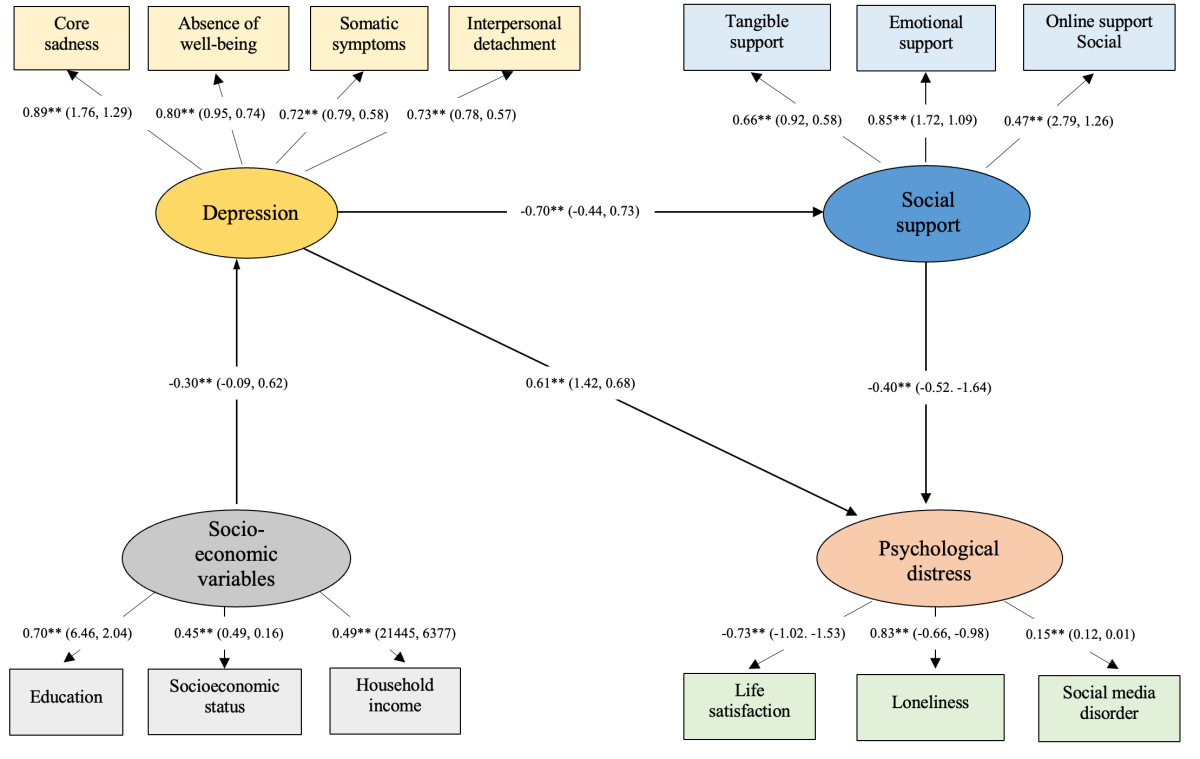
Construct validity of 12-Item Center for Epidemiological Studies Depression Scale (CES-D). The image shows standardized β values (maximum likelihood estimates), with the 95% CIs in parentheses. The * indicates *P*<.05 and the ** indicates *P*<.01.

Although lower socioeconomic status predicts depression, socioeconomic status did not predict social support or psychological well-being. It should also be noted that we relabeled psychological well-being as psychological distress to better reflect directions of association (ie, loneliness, life dissatisfaction, and SMU disorder; Figure 3).

Overall fit was ideal for this SEM model (*χ*^2^_56_=86.2; *P*<.01; CFI=0.97; SRMR=0.049; RMSEA=0.052; 0.028<RMSEA 90% confidence level<0.072). With 218 participants and 56 df, statistical power for this CFA model was sufficient to identify medium to large effect sizes (ie, *d*>0.80).

Depression as measured using this 4-factor CES-D significantly predicted lower social support and both directly and indirectly predicted psychological distress via lower social support. Both direct and indirect associations were statistically significant (Table 5). These findings support the construct validity of the responses to this revised 12-item CES-D.

**Table 5:** Direct and indirect effects of depression and social support.^a^

			Socioeconomic status	Depression		Social support
**Depression**						
	Direct effects	–0.30^c^		—^b^	—	
	Indirect effects	0.00^d^		—	—	
	Total effects	–0.30^c,d^		—	—	
**Social support**						
	Direct effects	0.00		–0.70^c^	—	
	Indirect effects	0.21^c,d^		0.00^d^	—	
	Total effects	0.2^c^		–0.70^c^	—	
**Psychological distress**						
	Direct effects	0.00		0.6^c^	–0.40^c^	
	Indirect effects	–0.27^c,d^		0.28^c,d^	0.00^d^	
	Total effects	–0.27^c^		0.89^c^	–0.40^c^	

^a^Statistical significance estimated over 500 bootstrapped samples.

^b^Not applicable.

^c^*P*<.01.

^d^Direct effects are added to indirect effects, sum = total effects.

## Discussion

### Principal Findings

For this study, we set out to devise a brief, online version of the CES-D with an online sample for contemporary digital research. As mental health research continues to transition to online platforms, there is a growing need for psychometric tools validated for online research [2,44]. Reported findings demonstrate that this 12-item CES-D preserves the established 4-factor structure of the original scale while offering more robust measurement of the interpersonal dimension of depression, an area particularly relevant in context of online social interaction (Multimedia Appendix 1).

Across analyses, responses to this 12-item CES-D demonstrated strong internal consistency and consistent support for the 4 first-order factors (core sadness, absence of well-being, somatic symptoms, and interpersonal detachment), each significantly measuring a higher-order depression latent factor. The construct validity of this online version of the CES-D was supported relative to social support, psychological distress, and socioeconomic indicators. Our findings underscore the central role of interpersonal processes in depression, particularly in the context of SMU and contemporary patterns of social support. The revised 12-item CES-D offers a brief yet psychometrically robust measure of depressive symptomatology, including its interpersonal dimension, providing efficient and sufficient assessment suitable for online research [28].

A central goal of this study was to strengthen the assessment of the interpersonal factor, reconceptualized as interpersonal detachment. Prior research has shown that 2-item factors can be psychometrically unstable, particularly when used with diverse or nonclinical online samples. Our results confirm that expanding interpersonal measurement improves the reliability and factorial stability of the scale while maintaining the brevity essential for online administration. These findings are consistent with long-standing evidence that interpersonal difficulties, including loneliness, social withdrawal, and perceived rejection, are integral components of depressive symptomatology and not merely peripheral correlates.

Multivariate analyses suggest that social support serves as a buffer, mitigating the adverse effects of depression on psychological distress. Socioeconomic factors predicted depression and indirectly impacted psychological distress via depression. Educational level, socioeconomic status based on work performed, and household income collectively and uniquely contributed to measurement of socioeconomic status. In contrast, age, gender, and employment status did not significantly contribute to measurement, leading us to relabel this latent factor as socioeconomic status, not sociodemographic status. This suggests that economic factors such as income are more germane to depression than sociodemographic variables such as age. At least this appears true for relatively low-income participants. The average household income of the participants (both groups) at US $74,920 (SD US $55,478) is below the US median of US $83,730 for 2024 [45].

Consistent with our focus on digitally mediated social experience, we extended measurement of traditional social support (emotional and tangible assistance) to include online social support. Each contributed uniquely and significantly to the measurement of social support, underscoring the need to account for both offline and online relational resources in contemporary research. In addition, for this study, psychological distress was operationalized to include symptoms of SMU disorder in accordance with emerging evidence suggesting that problematic social media engagement may intersect with depressive processes [46]. Although loneliness and life dissatisfaction were the strongest indicators, findings underscore that social media functions both as a resource for connection and a source of strain for adults with depression [47].

This is not the first depression scale devised for online research. For instance, the 12-item Mental Health Screening Tool for Depressive Disorders proposed by Park et al [48] was developed to screen for depression in South Korea. (The CES-D was used in their study to demonstrate the concurrent validity of Mental Health Screening Tool for Depressive Disorders responses.) Similarly, Loe et al [14] described the development of a 17-item version of the CES-D [14]. However, 1 of the 3 items they suggested be deleted was an interpersonal detachment item, reducing measurement to a single item. In addition, a reduction of 3 items is not sufficient to consider this a brief version of the CES-D.

### Limitations and Future Research

While this study reports notable findings, several limitations must also be acknowledged. First, as previously noted, the CES-D is widely used as both a screening tool and measure of the phenomenology of depression [12,13,17,25,49,50]; we do not address the former. Future research is needed to ascertain the scores suggestive of clinical symptomology for this 12-item online version of the CES-D [51,52].

Although establishing a clinical cutoff was not the focus of this study, research with existing versions of the scale provide a basis for estimation. Specifically, if we interpolate between the established cutoff for the 10-item CES-D (>10/30 [53]) and the original 20-item CES-D (>15/30 [29]), scores greater than 11/36 on the 12-item CES-D may suggest elevated depressive symptom levels. Clinical research is required in the future to ascertain the sensitivity and specificity of this and other cutoff points.

A second limitation is the cross-sectional design of this study, which prevents us from drawing causal associations among depressive symptoms, social support, and psychological distress. Associations are likely bidirectional over time [54]. Longitudinal research is needed to better understand how these variables interact. This will enable estimation of test-retest reliability, measurement invariance across time across factors, and differential change in depressive symptoms (eg, depressive subtypes). For instance, researchers could examine how various trajectories of depressive symptoms as measured using this 12-item CES-D impact mental health and well-being. Do different CES-D factors differentially impact psychological distress over time?

It is also important to stress that depression diagnoses were self-reported by participants and not clinically corroborated (eg, structured clinical interviews), nor did we collect information about treatment history, including psychiatric hospitalizations. However, CES-D scores were high in this sample, suggestive of clinical symptomology [55]. In addition, as previously noted with participants with depression and those with bipolar disorder [56], participants recruited online and via social media also appear more symptomatic than traditional samples composed of psychiatric outpatients. Future research is needed to determine how healthy and problematic SMU differ across mental health conditions (eg, social anxiety vs agoraphobia).

### Conclusions

This study introduces a concise, psychometrically robust 12-item online CES-D that more fully measures interpersonal aspects of depression, an increasingly salient component of mood disturbance in digitally networked societies. Scale responses demonstrated strong factorial integrity, reliability, and construct validity with participants with both depression and euthymia recruited online. As online mental health research continues to expand, this online version of the CES-D provides an efficient measure suitable for use across diverse digital research settings. Continued validation across clinical, community, and cross-cultural samples will further clarify the scale’s utility and ensure its relevance in the evolving landscape of mental health assessment.

## Appendix

Multimedia Appendix 1 Brief, online version of the Center for Epidemiological Studies Depression Scale (CES-D).

## Abbreviations

CES-D: Center for Epidemiological Studies Depression Scale
CFA: confirmatory factor analysis
CFI: comparative fit index
ECVI: expected cross-validation index
RMSEA: root mean square error of approximation
SEM: structural equation modeling
SLS: Satisfaction With Life Scale
SMD: Social Media Disorder Scale
SMU: social media use
SRMR: standardized root mean square residual

## Data Availability

Anonymized datasets analyzed during this study are available in the Harvard Dataverse repository [57].
