# Supplementary material for: Development and Initial Validation of a Brief, Online Version of the Center for Epidemiological Studies Depression Scale (CES-D): Psychometric Study
Source: JMIR Form Res. 2026 Jan 8;10:e81595. doi: 10.2196/81595 (PMC12828309; doi:10.2196/81595)
Supplement: Multimedia Appendix 1 [file formative_v10i1e81595_app1.docx]

**Appendix 1**

Center for Epidemiologic Studies – Depression (CES-D) Scale

For each of the following statements, please indicate how often you felt this way **during the past week** using the response key above.

 ---------------------------------------------------------------------------------------------------------------

**0 1 2 3**

Rarely or none [< 1 day] Some [1-2 days] Occasionally [3-4 days] Most [5-7 days]

 ---------------------------------------------------------------------------------------------------------------

1. I was bothered by things that don’t usually bother me **0 1 2 3**

2. I did not feel like eating; my appetite was poor **0 1 2 3**

3. I felt that I could not shake off the blues even with help from my family **0 1 2 3**

or friends

4. I felt that I was just as good as other people **3 2 1 0**

5. I had trouble keeping my mind on what I was doing **0 1 2 3**

6. I felt depressed **0 1 2 3**

7. I felt that everything I did was an effort **0 1 2 3**

8. I felt hopeful about the future **3 2 1 0**

9. I thought my life had been a failure **0 1 2 3**

10. I felt fearful **0 1 2 3**

11. My sleep was restless **0 1 2 3**

12. I was happy **3 2 1 0**

13. I talked less than usual **0 1 2 3**

14. I felt lonely **0 1 2 3**

15. People were unfriendly **0 1 2 3**

16. I enjoyed life **3 2 1 0**

17. I had crying spells **0 1 2 3**

18. I felt sad **0 1 2 3**

19. I felt that people dislike me **0 1 2 3**

20. I could not *get going* **0 1 2 3**

Absence of Well-Being CES08, CES12, CES16 (reverse keyed)

Somatic Symptoms CES07, CES13, CES20

Interpersonal Detachment CES14, CES15, CES19

Core Sadness CES03, CES06, CES18
